# Perineal trauma after vaginal delivery in healthy pregnant women

**DOI:** 10.1590/1516-3180.2014.1324710

**Published:** 2014-05-28

**Authors:** Larissa Santos Oliveira, Luiz Gustavo Oliveira Brito, Silvana Maria Quintana, Geraldo Duarte, Alessandra Cristina Marcolin

**Affiliations:** I MD. Physician, Department of Gynecology and Obstetrics, Faculdade de Medicina de Ribeirão Preto (FMRP), Universidade de São Paulo (USP), Ribeirão Preto, São Paulo, Brazil; II MD, PhD. Attending Physician, Department of Gynecology and Obstetrics, Faculdade de Medicina de Ribeirão Preto (FMRP), Universidade de São Paulo (USP), Ribeirão Preto, São Paulo, Brazil; III MD, PhD. Associate Professor, Department of Gynecology and Obstetrics, Faculdade de Medicina de Ribeirão Preto (FMRP), Universidade de São Paulo (USP), Ribeirão Preto, São Paulo, Brazil; IV MD, PhD. Full Professor, Department of Gynecology and Obstetrics, Faculdade de Medicina de Ribeirão Preto (FMRP), Universidade de São Paulo (USP), Ribeirão Preto, São Paulo, Brazil; V MD, PhD. Professor, Department of Gynecology and Obstetrics, Faculdade de Medicina de Ribeirão Preto (FMRP), Universidade de São Paulo (USP), Ribeirão Preto, São Paulo, Brazil

**Keywords:** Lacerations, Labor stage, second, Postural balance, Episiotomy, Obstetrical forceps, Lacerações, Segunda fase do trabalho de parto, Equilíbrio postural, Episiotomia, Forceps obstétrico

## Abstract

**CONTEXT AND OBJECTIVE:**

Despite all the medical care provided during delivery labor, perineal injury is still prevalent and may lead to diverse pelvic floor disorders. The aim here was to investigate the prevalence of obstetric and anal sphincter injuries (OASIS) in healthy pregnant women after vaginal delivery.

**DESIGN AND SETTING:**

Cross-sectional study involving 3,034 patients with singletons in a secondary hospital for low-risk cases.

**METHODS:**

A standardized questionnaire was prepared and applied to medical files that had been completely filled out (classification of the Royal College of Obstetricians and Gynecologists, RCOG) in order to identify OASIS and analyze risk factors associated with mild and severe perineal lacerations.

**RESULTS:**

The women's mean age was 25 years; more than half (54.4%) were primiparae. Almost 38% of the participants had perineal lacerations; these were severe in 0.9% of the cases. Previous vaginal delivery (odds ratio, OR: 1.64 [1.33-2.04]) and forceps delivery (OR: 2.04 [1.39-2.97]) were risk factors associated with mild perineal injuries (1^st^ and 2^nd^ OASIS classifications). Only remaining standing for prolonged periods during professional activity (OR: 2.85 [1.34-6.09]) was associated with severe perineal injuries.

**CONCLUSION:**

The prevalence of severe perineal injuries was concordant with data in the literature. The variable of standing position was considered to be a risk factor for severe perineal injury and should be further investigated.

## Abstract (pt)

**CONTEXTO E OBJETIVOS:**

Apesar do cuidado médico executado durante o trabalho de parto, os traumas perineais ainda são prevalentes e podem levar a várias desordens do assoalho pélvico. O objetivo foi investigar a prevalência de injúrias obstétricas e do esfíncter anal em mulheres saudáveis após parto vaginal.

**DESENHO E LOCAL DE ESTUDO:**

Estudo transversal envolvendo 3.034 pacientes com recém-natos únicos de um hospital secundário de baixo risco.

**MÉTODOS:**

Um questionário padronizado foi preparado e aplicado aos prontuários completamente preenchidos (classificação do Royal College of Obstetricians and Gynecologists) para identificar as lesões obstétricas e do esfíncter anal e analisar fatores de risco associados com lacerações perineais leves e graves.

**RESULTADOS:**

A média de idade das mulheres era 25 anos; mais da metade (54,4%) era primípara. Quase 38% das participantes tiveram lacerações perineais; estas foram graves em 0,9% dos casos. A presença de parto vaginal prévio (odds ratio, OR, 1,64 [1,33-2,04]) e o parto fórceps (OR 2,04 [1,39-2,97]) foram fatores de risco associados às lesões perineais leves (primeira e segunda classificações de lesão esfíncter e anal). Somente a posição em pé prolongada durante a atividade profissional (OR 2,85 [1,34-6,09]) estava associada com lesões perineais graves.

**CONCLUSÃO:**

A prevalência de trauma perineal grave concordou com dados da literatura. A variável posição em pé foi considerada fator de risco para trauma perineal grave e necessita ser investigada.

## INTRODUCTION

Perineal tears may occur after vaginal delivery. This event may lead to fecal and urinary incontinence, chronic pelvic pain and dyspareunia in young women.^1^Epidemiological studies have demonstrated that perineal trauma correlates with maternal, fetal and birth delivery factors.^1^ Classifications for these complications have been devised1 and they have been named obstetric and anal sphincter injuries (OASIS) by urogynecologists and obstetricians.

However, the literature lacks appropriate classification systems that would predict the risks of perineal tears during obstetric care.^2^ Advanced maternal age,^3^ ^,^ ^4^ white color,^5^ primiparity^6^ ^,^ ^7^ and obesity^8^ seem to be perineum-related risk factors. Heavier birth weight^4^ ^,^ ^7^ and persistent occipitoposterior position^9^ are among the fetal risk factors for perineal tears. The birth factors of prolonged second stage of labor,^7^ ^,^ ^10^ birth analgesia,^11^ ^,^ ^12^ episiotomy^13^ and assisted vaginal delivery^4^ ^,^ ^6^ ^,^ ^7^ increase the risk of perineal trauma. 

However, it is unclear whether professional activity, administration of oxytocin and misoprostol during birth delivery and length of ruptured membranes constitute risk factors for perineal tears. Despite the current knowledge about the risk factors for severe perineal trauma, these factors depend on the type of laceration. However, investigations correlating maternal morbidity with obstetric perineal trauma are scarce. 

## OBJECTIVE

This study aimed to calculate the prevalence of obstetric and anal sphincter injuries during vaginal deliveries; to identify maternal, obstetric and fetal risk factors associated with perineal tears after vaginal deliveries; and to assess immediate maternal and early neonatal morbidities.

## METHODS

This was a cross-sectional study that evaluated patients attended at Centro de Referência da Saúde da Mulher de Ribeirão Preto (MATER), Ribeirão Preto, São Paulo, Brazil, during 2009 and 2010. The local Ethics Committee approved this investigation, which was conducted in accordance with an institutional protocol (Department of Gynecology and Obstetrics, Ribeirão Preto School of Medicine, University of São Paulo). This hospital is a training center for residents in Obstetrics and Gynecology. Deliveries are performed by medical students and first and second- year medical residents supervised by attending physicians.

Three thousand and thirty-four women were included. The inclusion criteria were that they should have singletons with gestational age > 37 weeks (determined according to the last menstruation period and confirmed through obstetric ultrasound), with cephalic fetal presentation, born by means of vaginal delivery and whose data were entirely recorded in the reports. The exclusion criteria were maternal diseases (hypertension, endocrine disorders or neurological disorders), pregnancies with fetal malformation and deliveries performed outside the hospital. A questionnaire was elaborated by two physicians, in order to collect data from these women's medical files, and it was applied by a medical student.

The patients were divided into three groups: (I) patients who did not present any delivery traumas; (II) patients who presented first and second-degree (mild) perineal tears; and (III) patients who presented third and fourth-degree (severe) perineal tears. The patients' records and information about the newborns were obtained from an institutional databank. Variables relating to the patient, newborn, birth and postpartum period were obtained directly from the patient's medical records. 

On the basis of a sample consisting of at least 710 patients per group, at a 5% significance level and 80% power, we estimated that the incidence of OASIS among women with a previous history of vaginal birth was 15% and 3% in primiparae and multiparae, respectively. We analyzed the following variables: (a) maternal factors: age, ethnicity, predominant posture while carrying out professional activity (standing position during work, sitting position etc.), use of tobacco and/or illicit drugs, nutritional status (maternal height and BMI) and previous vaginal birth; (b) obstetric factors: use of misoprostol to induce birth, use of oxytocin during the first and second stages of labor, length of membrane rupture, duration of second stage, presence of mediolateral episiotomy, assisted vaginal birth (forceps/vacuum) and use of locoregional analgesics; (c) fetal factors: fetal position at time of fetal expulsion, weight, skull circumference and gender of the newborn. 

Perineal trauma was classified into four degrees, according to the extent of perineal and anal canal damage, following the protocol of the Royal College of Obstetricians and Gynecologists (RCOG, 2001):^1^ first-degree trauma was limited to the vaginal mucosa and perineal skin, second-degree trauma included the perineal musculature, third-degree trauma involved the anal sphincter, and fourth-degree trauma included the rectal mucosa. 

To evaluate maternal morbidity relating to perineal trauma after birth delivery, the following parameters were considered: massive blood loss with hemodynamic instability (demanding blood transfusion or surgical approach), postpartum infection (endometritis, abscess or perineal wound infection), perineal wound dehiscence and length of hospital stay. To assess the newborns' conditions, the Apgar index, amniotic fluid conditions (presence or absence of meconium), mechanical ventilation, use of pressure support, admission to the neonatal intensive therapy unit and neonatal deaths were considered. 

Using the PROC LOGISTIC program of the SAS 9.0 software (SAS Institute Inc, Cary, NC, USA), the initial statistical analysis was performed by means of the chi-square test. Simple and multiple logistic regression methods were used to estimate the odds ratios when the independent variable studied was binomial. Multiple and simple multinomial logistic regression was used to estimate the odds ratio when the independent variable had more than two categories. This part of the statistical analysis was conducted with the aid of the PROC CATMOD program of the SAS 9.0 software. 

## RESULTS

Out of a total of 3,425 records evaluated, 3,034 women fulfilled the inclusion criteria for this study: 1,650 women (54.4%) had had previous vaginal deliveries and 1,384 (45.6%) were having their first child. We found occurrences of mild and severe perineal trauma in 1,105 (36.42%) and 27 (0.9%) of the patients, respectively. Among the patients with previous vaginal births, 771 (46.7%) presented lacerations: 759 (98.4%) had first and second-degree lacerations and 12 (1.6%) had third and fourthdegree lacerations. Among the primiparous women, 361 (26.1%) presented perineal tears: 346 (95.8%) presented first and second-degree tears and 15 (4.2%) presented third and fourth-degree tears.

Table 1 presents the maternal, obstetric and neonatal variables of the population studied and their correlations with lacerations. The following variables influenced the type of laceration: the maternal variables of age, body position during professional activity, body mass index (BMI) and at least one previous vaginal delivery; the obstetric variables of use of oxytocin, time of membrane rupture, duration of the second period, episiotomy, locoregional analgesia and assisted vaginal delivery; and the neonatal variable of cranial circumference. The other variables did not affect the degree of laceration significantly. Table 2 shows the relative risk (as odds ratio values, OR) of laceration (any type), in relation to the maternal, obstetric and neonatal variables, after application of a multiple logistic regression model. 

**Table 1:** Risk of perineal trauma presented by the variables studied

	Perineal trauma						P-value
	None (%)		Mild (%)		Severe (%)		
Age (mean ± standard deviation; years)	23.41 ± 8.9		24.87 + 6.9		25.14 ± 7.0		< 0.0001
≤ 19	29.4		20.0		25.9		
20-35	66.8		73.7		59.3		
> 35	3.8		6.3		14.8		
Race							
White	65.2		63.5		52.9		> 0.05
Body position during professional activity							
Standing	18.9		20.0		40.7		0.0149
Sitting	81.1		80.0		59.3		
Use of tobacco and/or illicit drug							
Yes	15.1		15.7		11.1		> 0.05
No	84.9		84.3		88.9		
Body mass index							
Normal	63.4		55.1		65.0		0.0024
Overweight	23.0		27.4		25.0		
Obese	13.6		17.5		10.0		
Maternal height (m)							
< 1.50	3.2		2.8		5.0		> 0.05
1.50-1.55	18.6		19.4		35.0		
1.56-1.60	29.7		28.6		35.0		
1.61-1.65	28.9		29.6		20.0		
> 1.65	19.6		19.6		5.0		
Previous vaginal delivery							
Yes	46.2		68.7		44.4		< 0.0001
No	53.8		31.3		55.6		
Use of oxytocin							
Yes	59.0		53.8		55.6		0.0183
No	41.0		46.2		44.4		
Use of misoprostol							
Yes	17.5		18.3		29.6		> 0.05
Length of time with ruptured membranes (h)							
< 6	78.9		82.2		52.2		0.01
6-12	11.8		10.6		26.1		
12-18	5.4		4.0		13.0		
> 18	3.9		3.2		8.7		
Duration of the second period (min)							
< 30	85.0		92.6		85.0		< 0.0001
30-60	9.2		5.1		5.0		
> 60	5.8		2.3		10.0		
Episiotomy							
Yes	58.8		4.9		29.6		< 0.0001
No	41.2		91.1		70.4		
Locoregional analgesia							
Yes	67.3		56.2		67.3		< 0.0001
No	32.7		43.8		32.7		
Assisted vaginal delivery							
Yes	9.1		2.3		14,8		< 0.0001
No	90.9		97.7		85,2		
Fetal presentation							
Flexed head	90.7		91.5		92.6		> 0.05
Deflexed head	9.3		8.5		7.4		
Birth weight (g)							
< 2700	12.5		10.0		12.5		> 0.05
2700-3800	80.0		80.5		70.8		
Cranial circumference (cm)							
≤ 33		32.8		26.2		25	0.002
> 33		67.2		73.8		75	

**Table 2:** Relative risk of perineal trauma among the variables studied, after multivariate analysis

	Crude		Adjusted	
	Odds ratio	95% CI	Odds ratio	95% CI
Maternal age (years)				
≤ 19 versus 20- 35	0.46	(0.25-0.85)	1.10	(0.51-2.39)
20-35 versus > 35	0.74	(0.41-1.32)	0.86	(0.43-1.72)
Body position during professional activity				
Standing versus sitting	1.16	(0.89-1.51)	1.01	(0.72-1.41)
Body mass index				
Obese versus normal	1.32	(0.98-1.78)	1.05	(0.72-1.55)
Overweight versus normal	1.26	(0.98-1.62)	1.12	(0.81-1.54)
Previous vaginal delivery				
No versus yes	0.33	(0.27-0.42)	2.67	(1.75-4.10)
Use of oxytocin				
Yes versus no	0.69	(0.56-0.86)	1.06	(0.74-1.52)
Length of time with ruptured membranes (h)				
6-12 versus < 6	0.91	(0.66-1.27)	1.19	(0.76-1.86)
12-18 versus < 6	0.70	(0.41-1.20)	0.51	(0.25-1.05)
> 18 versus < 6	0.98	(0.56-1.71)	1.37	(0.62-3.03)
Second-stage duration (min)				
30-60 versus > 60	1.80	(0.81-4.01)	1.08	(0.39-3.02)
< 30 versus > 60	3.39	(1.70-6.76)	1.22	(0.50-2.97)
Episiotomy				
No versus yes	26.03	(18.13-37.37)	88.96	(50.53-156.61)
Locoregional analgesia				
No versus yes	2.02	(1.61-2.54)	1.10	(0.79-1.53)
Assisted vaginal delivery				
Yes versus no	0.28	(0.17-0.48)	4.48	(2.20-9.11)
Cranial circumference (cm)				
> 33 versus ≤ 33	1.31	(1.03-1.65)	(1.03-1.65)	(0.81-1.58)

CI = confidence interval

Multivariate analysis revealed that maternal age, position maintained during professional activity, BMI, use of oxytocin during the active phase of labor, time of membrane rupture, duration of the second period, locoregional analgesia and neonatal cranial circumference were not risk factors for perineal trauma. Primiparity, episiotomy and assisted vaginal delivery presented 2.7, 89 and 4.5-fold increased risk of perineal trauma, respectively. 

Table 3 lists the relative risks of the maternal, obstetric and neonatal variables (as OR, obtained through multivariate logistic regression) for first and second-degree perineal tears. Multivariate analysis showed that maternal age, position maintained during professional activity, BMI, use of oxytocin during the active phase of labor, duration of the second stage, locoregional analgesia, neonatal cranial circumference and birth weight were not risk factors for first and second-degree perineal tears. Primiparity, nonuse of episiotomy and use of assisted vaginal delivery presented 1.6, 10 and 2-fold higher risk of first and second-degree perineal trauma, respectively. Interestingly, membrane rupture that occurred between 12 and 18 h before delivery reduced the risk of perineal tears by almost 50% (OR 0.54, 95% confidence interval, CI: 0.31-0.94). 

**Table 3:** Relative risk of mild perineal trauma among the variables studied, after multivariate analysis

	Crude		Adjusted	
	Odds ratio	95% CI	Odds ratio	95% CI
Maternal age (years)				
≤ 19 versus 20- 35	0.63	(0.54- 0.74)	1.12	(0.79-1.57)
20-35 versus > 35	1.03	(0.90- 1.17)	0.87	(0.66-1.15)
Body position during professional activity				
Standing versus sitting	1.04	(0.94-1.14)	0.96	(0.81-1.14)
Body mass index				
Obese versus normal	1.17	(1.00-1.37)	1.01	(0.79-1.30)
Overweight versus normal	1.08	(0.95-1.24)	1.06	(0.85-1.31)
Previous vaginal delivery				
No versus yes	0.63	(0.58-0.68)	1.64	(1.33-2.04)
Use of oxytocin				
Yes versus no	0.90	(0.83-0.97)	1.03	(0.86-1.23)
Length of time with ruptured membranes (h)				
6-12 versus < 6	1.03	(0.83-1.29)	1.16	(0.77-1.75)
12-18 versus < 6	0.85	(0.63-1.14)	0.54	(0.31-0.94)
> 18 versus < 6	0.94	(0.68-1.30)	1.47	(0.79-2.71)
Second-stage duration (min)				
30-60 versus > 60	0.89	(0.68-1.17)	1.00	(0.61-1.65)
< 30 versus > 60	1.77	(1.45-2.16)	1.13	(0.77-1.68)
Episiotomy				
No versus yes	5.27	(4.57- 6.09)	9.93	(7.41-13.31)
Locoregional analgesia				
No versus yes	1.27	(1.17-1.37)	1.05	(0.88-1.24)
Assisted vaginal delivery				
Yes versus no	0.48	(0.39-0.60)	2.04	(1.39-2.97)
Cranial circumference (cm)				
> 33 versus ≤ 33	1.17	(1.07-1.29)	1.06	(0.90-1.26)
Birth weight (g)				
2700-3800 versus < 2700	1.00	(0.87-1.15)	1.03	(0.90-1.26)
> 3800 versus < 2700	1.25	(1.02-1.54)	1.01	(0.95-1.49)

CI = confidence interval

Table 4 summarizes the relative risks of the maternal, obstetric and neonatal variables for third and fourth-degree perineal traumas. None of the statistically significant variables for perineal trauma shown in Table 1 remained as risk factors after adjusted logistic regression. The exception was body position during professional activity, since pregnant women who remained in a standing position most of the time had a threefold higher risk of perineal trauma (OR 2.85; 95% CI: 1.34-6.09). Table 5 shows the maternal and perinatal results. First and fifth-minute Apgar scores less than 7, meconium fluid present at the time of delivery, mechanical ventilation, neonatal death, bleeding disorders and postpartum maternal infection did not significantly impact the degree of laceration. We were unable to correlate dehiscence of episiotomy sutures with the degree of perineal laceration. No maternal deaths occurred among this sample. Patients with severe perineal trauma were at increased risk of remaining in the hospital for more than four days, compared with patients presenting mild or no perineal trauma. 

**Table 4:** Relative risk of severe perineal trauma among the variables studied, after multivariate analysis

	Crude		Adjusted	
	Odds ratio	95% CI	Odds ratio	95% CI
Maternal age (years)				
≤ 19 versus 20-35	0.61	(0.33-1.14)	0.96	(0.24-3.92)
20-35 versus > 35	0.61	(0.36-1.04)	0.62	(0,21-1.79)
Body position during professional activity				
Standing versus sitting	1.72	(1.17-2.53)	2.85	(1.34-6.09)
Body mass index				
Obese versus normal	0.78	(0.29-2.11)	0.66	(0.14-3.07)
Overweight versus normal	1.16	(0.54-2.52)	1.09	(0.33-3.55)
Previous vaginal delivery				
No versus yes	1.04	(0.71-1.52)	1.37	(0.48-3.95)
Use of oxytocin				
Yes versus no	0.93	(0.64-1.36)	1.45	(0.41-5.19)
Length of time with ruptured membranes (h)				
6-12 versus < 6	1.32	(0.61-2.85)	2.93	(0.90-9.56)
12-18 versus < 6	1.43	(0.55-3.75)	0.95	(0.14-6.59)
> 18 versus < 6	1.34	(0.44-4.11)	0.91	(0.13-6.25)
Second-stage duration (min)				
30-60 versus > 60	0.55	(0.14-2.24)	0.74	(0.13-4.21)
< 30 versus > 60	1.02	(0.43-2.43)	1.19	(0.32-4.38)
Episiotomy				
No versus yes	1.84	(1.22- 2.79)	2.60	(0.84-7.99)
Locoregional analgesia				
No versus yes	0.93	(0.61-1.41)	0.99	(0.40-2.46)
Assisted vaginal delivery				
Yes versus no	1.32	(0.77-2.25)	3.07	(0.98-9.60)
Cranial circumference (cm)				
> 33 versus ≤ 33	1.21	(0.76-1.93)	1.16	(0.50-2.68)
Birth weight (g)				
2700-3800 versus < 2700	0.71	(0.39-1.28)	0.64	(0.19-2.11)
> 3800 versus < 2700	1.77	(0.81-3.87)	0.42	(0.95-20.57)

CI = confidence interval.

**Table 5:** Maternal and neonatal outcomes from the population studied

	Perineal trauma			P-value
	None (n = 1,902)	Mild (n = 1,105)	Severe (n = 27)	
Neonatal variables (%)				
1st minute Apgar < 7	11.3	9.8	11.1	0.47
5th minute Apgar < 7	0.9	1.18	3.7	0.30
Meconium fluid	13.2	13.3	7.4	0.72
Ventilatory support	2.0	1.7	3.7	0.69
Neonatal death	0.3	0.3	0	0.50
Maternal variables (%)				
Postpartum hemorrhage	1.1	0.9	0	0.70
Postpartum infection	0.7	0.9	3.7	0.18
Hospitalization duration > 4 days	19.3	15.6	29.6	0.01

## DISCUSSION

We found that the prevalence of severe perineal tears (third and fourth-degree) was 0.9% in our cohort, which can be considered low in comparison with some previous studies^14^ ^,^ ^15^ but similar to or higher than values reported from other samples.^16^ ^,^ ^17^ For example, one study in the United States found a prevalence of severe laceration of 0.25%.^18^ Lack of a national database in Brazil prevents comparison of our results with those from other Brazilian regions. Misdiagnosis of perineal tears associated with inexperience among surgeons and inadequate surgical repair during the postpartum period may affect the prevalence of severe perineal trauma. Previous studies^19^ ^,^ ^20^ have shown that obstetrics-gynecology residents cannot identify OASIS properly, and therefore underreporting of these events may influence the recorded laceration patterns.

On the other hand, better prenatal care, ultrasonographic diagnosing of macrosomic fetuses, manual support of the perineum and selective use of mediolateral episiotomy (MLE) could explain the low incidence of perineal tears. Multivariate analysis in the present study revealed that the variable of MLE had a protective effect in relation to mild and severe perineal trauma, which means that it is possible for MLE to be used as a perineal protective maneuver during the second period of labor. However, the data on MLE in the literature are divergent. A retrospective study on 3,038 deliveries in a British hospital also showed that MLE was a protective factor against severe perineal trauma,^15^ but another retrospective study including a larger number of deliveries (n = 168,077) found the opposite result^21^ and did not recommend prophylactic use of MLE. A Scandinavian group obtained different results when they separated 514,741 cases between primiparous and multiparous women: MLE was a protective factor for the former and a risk factor for the latter.^22^

One atypical finding from the present study was that the standing position during occupational activity is a risk factor for third and fourth-degree perineal trauma. Different body positions impact evaluations on the pelvic floor differently: basal vaginal pressure is higher while standing than while lying down.^23^ This affects the reliability of assessments on genital prolapse, because two-digit palpation correlates with prolapse symptoms when patients are examined in the standing position.^24^ Regarding pregnancy, associations between occupational activities (long working hours and prolonged standing periods) and unfavorable outcomes (prematurity and intrauterine growth restriction) are still a matter of controversy.^25^ Compared with the supine position, adopting a standing position during labor does not increase the risk of perineal trauma.^26^ To our knowledge, this variable has never been analyzed as an independent risk factor for perineal trauma. Studies on pelvic anatomy during and after birth labor have focused on assessment of changes that happen during the procedure; however, they have seldom evaluated factors that precede birth labor. Further studies are important for elucidating this issue. 

We found an association between primiparity and mild perineal trauma. Several studies have reported a relationship between the first delivery and perineal tears.^27^ ^,^ ^28^ High pelvic muscle tone (not previously tested) may reduce vulvovaginal elasticity during the first and second labor stages, thus increasing the risk of perineal lesions. 

We also observed that assisted vaginal delivery correlated with mild perineal trauma. Use of forceps may culminate in severe perineal trauma during fetal extraction, and so the obstetrician must be careful and experienced. Vacuum extraction delivery seems to lead to less perineal trauma than does forceps delivery.^27^

A length of time with ruptured membranes of between 12 and 18 h plays a protective role in relation to mild perineal trauma. It is hard to explain this finding, because preterm rupture of membranes results in birth labor that demands more analgesia, which in turn lessens the hydraulic cushion exerted by the amniotic fluid and impacts the pelviperineal region.^29^

The strengths of this study were its large sample, the great diversity of fetal-maternal variables and the possibility of analyzing new epidemiological variables (e.g. the standing position) that may influence future recommendations for preventing OASIS. The limitations of this study were its retrospective nature and the possibility that the professionals investigating OASIS might have underreported it. Estimates have shown that occult OASIS occurs in approximately 20-30% of deliveries, and this should be discussed when healthcare professionals are being trained.

Finally, more prospective studies are necessary, in order to assess the risk factors associated with mild and severe perineal trauma. Standing position is a new risk factor that should be investigated in future studies. Further studies comparing types of episiotomy should be conducted, because of the findings that the mediolateral type has a protective role. It is important to identify women who are at risk of OASIS during birth labor, in order to minimize the risks of perineal trauma during this period. Obstetricians need to have advanced knowledge of pelviperineal anatomy, so as not to cause harm during labor. 

## CONCLUSIONS

The prevalence of perineal tears found in this study was concordant with data in the literature. The standing position was a risk factor for severe perineal trauma. Episiotomy presented a protective role, in comparison with other variables.
